# Perceived ability to perform daily hand activities after stroke and associated factors: a cross-sectional study

**DOI:** 10.1186/s12883-016-0733-x

**Published:** 2016-11-02

**Authors:** Elisabeth Ekstrand, Lars Rylander, Jan Lexell, Christina Brogårdh

**Affiliations:** 1 Department of Health Sciences, Lund University, Lund, Sweden; 2 Department of Hand Surgery, Skåne University Hospital, Malmö, Sweden; 3 Division of Occupational and Environmental Medicine, Lund University, Lund, Sweden; 4 Department of Neurology and Rehabilitation Medicine, Skåne University Hospital, Lund, Sweden; 5 Department of Health Science, Luleå University of Technology, Luleå, Sweden

**Keywords:** Activities of daily living, Association, Cross-sectional study, Rehabilitation, Stroke, Self report, Upper extremity

## Abstract

**Background:**

Despite that disability of the upper extremity is common after stroke, there is limited knowledge how it influences self-perceived ability to perform daily hand activities. The aim of this study was to describe which daily hand activities that persons with mild to moderate impairments of the upper extremity after stroke perceive difficult to perform and to evaluate how several potential factors are associated with the self-perceived performance.

**Methods:**

Seventy-five persons (72 % male) with mild to moderate impairments of the upper extremity after stroke (4 to 116 months) participated. Self-perceived ability to perform daily hand activities was rated with the ABILHAND Questionnaire. The perceived ability to perform daily hand activities and the potentially associated factors (age, gender, social and vocational situation, affected hand, upper extremity pain, spasticity, grip strength, somatosensation of the hand, manual dexterity, perceived participation and life satisfaction) were evaluated by linear regression models.

**Results:**

The activities that were perceived difficult or impossible for a majority of the participants were bimanual tasks that required fine manual dexterity of the more affected hand. The factor that had the strongest association with perceived ability to perform daily hand activities was dexterity (*p* < 0.001), which together with perceived participation (*p* = 0.002) explained 48 % of the variance in the final multivariate model.

**Conclusion:**

Persons with mild to moderate impairments of the upper extremity after stroke perceive that bimanual activities requiring fine manual dexterity are the most difficult to perform. Dexterity and perceived participation are factors specifically important to consider in the rehabilitation of the upper extremity after stroke in order to improve the ability to use the hands in daily life.

## Background

Disability of the upper extremity is common after stroke and almost 50 % of those affected have remaining impairments more than three months post-stroke [1, 2]. The impairments often lead to difficulties in performing daily hand activities [3], especially those that require the use of both hands, i.e., bimanual activities [4]. The ability to perform bimanual activities is therefore an important goal in stroke rehabilitation, regardless of which hand that is affected [5].

The ability to perform daily activities can be objectively assessed by observations of different tasks in a standardized environment or by patient-reported questionnaires. The advantage of using questionnaires is that they often provide a better understanding of an individual’s self-reported everyday difficulties and thereby enable clinicians to design more individually targeted rehabilitation interventions [6]. One questionnaire that is recommended for persons with disability of the upper extremity after stroke is the ABILHAND Questionnaire [4, 7, 8]. It assesses self-perceived ability to perform daily bimanual activities. Previous studies have focused on evaluating the psychometric properties of the ABILHAND [4, 8], but no study has thoroughly described which activities persons in a stable phase post stroke perceive difficult to perform.

In order to improve functioning of the upper extremity after stroke, it is important to understand which factors affect self-perceived ability to perform daily hand activities. Previous studies have shown that single factors, such as motor function, muscle strength, spasticity, somatosensation, dexterity, perceived participation and life satisfaction are moderately to strongly associated with the perceived ability [4, 9–17]. However, as several factors simultaneously may influence the ability to perform daily hand activities there is a need to understand how these factors are associated with the performance. To the best of our knowledge, only one study [14] has evaluated this association in persons in a stable phase after stroke. In that study by Harris and Eng [14], muscle strength, spasticity, somatosensation and pain were included in multivariate analyses and the authors found that muscle strength in the upper extremity and spasticity were the strongest contributing factors to the perceived ability to use the hands in daily activities. However, dexterity was omitted as a potentially associated factor in the analysis, which was addressed as a limitation of the study. In other studies, gender, dominance of the affected upper extremity, and social and vocational situations have been shown to be important factors for overall functioning after stroke [18–21]. However, it is unclear how these factors are associated with the self-perceived ability.

Taken together, despite that disability of the upper extremity is common after stroke there is limited knowledge of which daily activities that are perceived difficult to perform and which factors that affect the self-perceived performance. The majority of previous studies have evaluated how single or few factors are associated with perceived daily hand activities. Thus, there is a need for more studies that take several factors into account simultaneously.

The aim of this study was to evaluate a) which daily activities persons with mild to moderate impairments of the upper extremity after stroke perceive difficult to perform and b) how several factors (age, gender, social and vocational situation, affected hand, upper extremity pain, spasticity, grip strength, somatosensation, manual dexterity, perceived participation and life satisfaction) are associated with the self-perceived performance.

## Methods

### Participants

Persons diagnosed with stroke (ischemic or hemorrhagic) and admitted to the stroke unit at the Department of Neurology and Rehabilitation Medicine at Skåne University Hospital in the southern part of Sweden, were recruited from April 2012 to August 2015. They were identified through cooperation with physiotherapists and occupational therapists working in the stroke rehabilitation centres serving the acute neurology wards at Skåne University Hospital. The inclusion criteria were: (i) >18 years of age; ii) at least 4 months after stroke onset; (iii) mild to moderate impairments of the more affected upper extremity with preserved ability to take the palm to the forehead, and to grasp and release a small object. Persons were excluded if: (i) they were unable to follow instructions or (ii) had any other disorder or disease that affected the more affected upper extremity.

### Outcome measures

*Perceived ability to perform daily hand activities* was rated with the ABILHAND Questionnaire (stroke version) [4]. The ABILHAND consists of 23 common bimanual activities (see Table 2) that are rated as impossible (0 point), difficult (1 point) or easy (2 points). Items not attempted within the last three months are set as not applicable. The items are ordered hierarchically, from the most difficult items to the easiest, and they are also rated according to the level of bimanual involvement: A = breakable into unimanual sequences; B = requires stabilization with the more affected upper extremity; and C = requires fine bimanual dexterity [4]. The ABILHAND is Rasch analyzed [4], which means that ordinal data can be converted into an unidimensional interval scale, and presented in logits (i.e., log odds units) that ranges from plus to minus around zero as the center of the scale [22]. The higher the logit value, the better the self-perceived ability to use the upper extremities in daily hand activities. In this study, the Swedish version of the ABILHAND was used [23], which has been shown to have acceptable test-retest reliability for persons with mild to moderate impairments of the upper extremities after stroke [8]. Due to cultural differences the item *‘Peeling potatoes with a knife’* was changed to *‘Peeling potatoes’* (as a potato-peeler is commonly used in Sweden) and in the item *‘Tearing open a pack of chips’* the following words were added ‘*or a candy-bag’* (because older persons in Sweden more rarely eat chips) [8]. After the participants had responded to the ABILHAND the logits were obtained by entering the raw scores into an online data analysis module (http://www.rehab-scales.org/) established for chronic stroke patients [4].

*Pain* (present or not) was recorded by asking the participants if they perceived daily or almost daily pain in their more affected upper extremity.

*Spasticity* was assessed by the response to resistance of passive movement according to the Modified Ashworth Scale (MAS) [24]. The participants’ spasticity was assessed in an upright sitting position and was classified as present if the elbow, wrist or fingers had a score on the MAS larger or equal to 1 point. The MAS has been shown to have high intra-rater reliability of the upper extremity for persons with stroke [25].

*Grip strength* was measured with the digital dynamometer Grippit (Catell AB, Hägersten, Sweden, http://www.catell.se/). The Grippit is a portable device that is wirelessly connected to a computer. Grip strength was measured three times with the participants seated with the forearm supported on a table on a foam cushion (the shoulder in 30° flexion, the elbow in 90° flexion and the wrist in 0° to 15° dorsiflexion) according to a standardized test protocol [26]. Each contraction lasted 3 s with a 60 s rest interval between each repetition. The highest value in Newton (N) of the three contractions was recorded as the maximal grip strength. Measurements of grip strength with the Grippit dynamometer have been shown to have an acceptable test-retest reliability for persons with mild to moderate impairments of the upper extremity after stroke [26], and grip strength has also been found to be a representative measure of the entire upper extremity muscle strength after stroke [27].

*Active touch (*somatosensation) of the hand was assessed with the The Shape/Texture Identification test (STI-test) [28] (Össur Nordic AB, Uppsala, Sweden, http://www.ossur.se/). Active touch means that identification of different shapes and textures is done by active hand movements. Compared to passive touch, active touch has the advantage of reflecting how somatosensation is integrated during hand movements. The STI-test includes three shapes (cube, cylinder or hexagon) and three textures (one, two or three raised metal dots placed in a row) in three difficulty levels (decreasing sizes). According to the standardized test instructions [28], the participants were seated behind a screen and identified the shapes (presented randomly size for size) by the index finger. Thereafter the textures were presented and identified in the same way. The score of the STI-test ranges from 0 to 6 points per hand and a higher score indicates better somatosensation [28, 29]. The STI-test has been shown to have high test-retest reliability for persons with mild to moderate impairments of the upper extremity after stroke [29].

*Dexterity* was assessed by the modified Sollerman Hand Function test (mSHFT) [30] (Catell AB, Hägersten, Sweden, http://www.catell.se/). The mSHFT assesses manual dexterity by common pinch and volar grips. It is a short version of the 20-item original Sollerman Hand Function Test [31, 32] and consists of the three items most strongly correlated with the total score [30]. The items in the mSHFT are: number 4) picking up 4 coins of different sizes from a purse; number 8) putting 4 nuts in decreasing size on bolts; and number 10) buttoning 4 buttons in decreasing sizes. These items are performed as unimanual tasks and the ability to grasp the object correctly, the time to complete the item and the quality of the movement are both assessed on a 5-point scale (0 to 4 points). The total sum score ranges between 0 and 12 points for each hand (where 12 approximates normal dexterity) [30]. The mSHFT has been found to be valid and reliable for persons with mild to moderate impairments of the upper extremity after stroke [33].

*Perceived participation*, i.e., a person’s engagement in meaningful life situations, was rated by the participation domain of the Stroke Impact Scale 3.0 (SIS; Swedish version), that can be used as a separate scale [34, 35]. SIS Participation is interview-based and includes eight items: impact of stroke on work, social activities, quiet recreations, active recreations, role as a family member, religious activities, life control and ability to help others. The items are scored on a 5-point scale from 1 (limited all of the time) to 5 (limited none of the time). The mean for the items is calculated as a composite score and converted into a percentage value (from 0 to 100) [35], and a higher percentage value indicates higher perceived participation. SIS has been shown to be reliable and valid in persons with stroke [34, 36].

*Life Satisfaction* was rated by the Life Satisfaction Questionnaire (LiSat-11) [37]. LiSat-11 is interview-based and includes one item that assesses the level of global satisfaction with life as a whole and 10 items that assess the level of domain-specific satisfaction. In the present study, only the item of global satisfaction with life as a whole was used. The responses were rated on a six-graded scale: 6 = very satisfied; 5 = satisfied; 4 = rather satisfied; 3 = rather dissatisfied; 2 = dissatisfied; and 1 = very dissatisfied. In this study, the score was dichotomized into two categories; dissatisfied (score 1 to 4) and satisfied (5 and 6) according to Fugl-Meyer et al. [37].

### Procedures

Prior to the assessments the participants were asked about their age, handedness, social situation (if they lived alone or together with another person) and their vocational situation (not working or in work at least 20 h per week).

The assessments were performed in the following order: 1) perceived pain 2) ability to perform daily hand activities (ABILHAND) [4]; 3) spasticity (MAS) [24]; 4) perceived participation (SIS-Participation) [34]; 5) dexterity (mSHFT) [30]; 6) active touch (STI-test) [28]; 7) life satisfaction (LiSat-global satisfaction) [37]; and 8) grip strength (Grippit) [26]. Each test took about 10 min to complete and a short rest (approximately 5 min) was allowed between the tests. All assessments were performed on one occasion according to the standardized test procedures of each test in a quiet and separate room of the hospital by an experienced physiotherapist (first author). Data on time of stroke onset, type of stroke (ischemic or hemorrhagic) and side of paresis were verified from the medical records.

### Statistics

Descriptive statistics, such as frequencies, means and standard deviations (SD) and medians and minimum and maximum (min-max) were calculated for demographic and clinical characteristics of the participants, perceived ability to perform daily hand activities (logits) and potentially associated factors. The distribution of the participants’ ratings (easy, difficult, impossible or not applicable) of the 23 items in the ABILHAND was presented in percent.

Before the linear regression analyses were conducted correlations between the ordinal and continuous potentially associated factors were calculated using the Spearman correlation (rho) to investigate the strength of their associations.

Perceived ability to perform daily hand activities (continuous: logits) and the potentially associated factors were analyzed by linear regression models. The potentially associated factors were: age (continuous); gender (categorical: female *vs* male); social situation (categorical: living together with another *vs* living alone); vocational situation (categorical: working *vs* not working); affected hand (categorical; dominant *vs* non-dominant); perceived pain in the more affected upper extremity (categorical: present *vs* not present); spasticity in the more affected upper extremity (categorical: present *vs* not present); grip strength in the more affected hand (continuous: newton); active touch in the more affected hand (ordinal scale; 0 to 6 points); dexterity in the more affected hand (ordinal scale; 0 to 12 points), perceived participation (ordinal scale; 0 to 100 %) and life satisfaction (categorical: satisfied *vs* dissatisfied).

The multivariate regression building was made with a generous inclusion criterion (*p* ≤0.20) so that no potential variable was excluded in the early stages. First, the associations with perceived ability to perform daily hand activities were evaluated for one variable at the time. Secondly, the variable with the lowest *p*-value (if ≤0.20) was kept and thereafter the other variables were tentatively added, one at a time. Thirdly, the two variables with the lowest *p*-values (if both ≤0.20) were included and the remaining factors were tentatively added, one at a time. This procedure was continued as long as the *p*-values for all included factors were ≤0.20. This selection strategy, to evaluate the model at each step, was chosen as it increases the understanding of the different variables significance.

The explained variances (adjusted R^2^) after successive addition of the factors are given in the final multivariate model. To ensure the linearity, scatterplots were visually inspected for the bivariate associations. In addition, model assumptions were checked by means of residual analysis.

Data were analyzed with the IBM SPSS Statistics version 22 (IBM Corporation, Armonk, New York, United States).

## Results

### Demographics

**Fig. 1:**
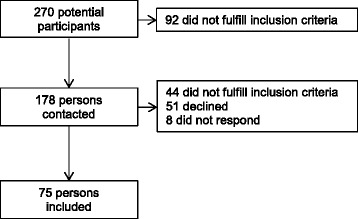
Study flowchart

Out of 270 potential participants, 75 persons with ischemic (*n* = 58) or hemorrhagic (*n* = 17) stroke were included in the study (Fig. 1 and Table 1). The mean time from stroke onset was 33 months (SD 26; range 4 to 116). The participants (72 % male) were on average 66 years (SD 8; range 44 to 85), one third lived alone and most of them did not work. About half of the participants were affected in their dominant hand.

**Table 1:** Characteristics of the 75 participants with stroke

Age, mean years (SD; range)	66 (8; 44 to 85)
Gender (male), n (%)	54 (72)
Time since stroke, mean months (SD; range)	33 (26; 4 to 116)
Stroke type, n (%)	
Ischemic	58 (77)
Hemorrhagic	17 (23)
Side of paresis (right), n (%)	37 (49)
Affected hand (dominant), n (%)	39 (52)
Social situation (living alone), n (%)	21 (28)
Vocational situation (not working), n (%)	62 (83)

### Description of perceived ability to perform daily hand activities

Table 2 shows the distribution of the ratings (ordinal data) of the 23 bimanual items in the ABILHAND in a hierarchical order. Between 41 and 61 % of the participants perceived the following eight items difficult or impossible to perform: ‘*filing one´s nails’; ‘hammering a nail’; ‘cutting meat’; wrapping up gifts’; ‘threading a needle’; ‘tearing open a pack of chips’; ‘buttoning up a shirt’; and ‘cutting one’s nails’*. All but one of these items except for ‘*buttoning up a shirt’* were classified as requiring fine manual dexterity of the more affected hand (level C) [4]. Thirteen items were perceived as easy to perform by most of the participants. Those that only required stabilization with the more affected hand (level B) were perceived as easy by 60 to 84 % of the participants, and those that could be breakable into unimanual sequences (level A) were perceived as easy by 63 to 95 % of the participants. Two of the items *‘shelling hazel nuts*‘and *‘sharpening a pencil’* (level C) were not attempted within the last 3 months for a majority of the participants and therefore noted as not applicable.

**Table 2:** Distribution (%) of ratings in the ABILHAND Questionnaire (*n* = 75)

	Items	Impossible	Difficult	Easy	NA	Level
1	Hammering a nail	19	28	31	23	C
2	Threading a needle	23	31	15	32	C
3	Peeling potatoes	13	35	51	1	C
4	Cutting one’s nails	19	42	39	0	C
5	Wrapping up gifts	16	35	23	27	C
6	Cutting meat	9	41	48	1	C
7	Filing one’s nails	8	33	31	28	C
8	Peeling onions	12	29	51	8	C
9	Shelling hazel nuts	0	9	20	71	C
10	Opening a screw-topped jar	5	23	71	1	C
11	Fastening the zipper of a jacket	3	37	60	0	B
12	Tearing open a pack of chips	15	41	40	4	C
13	Buttoning up a shirt	12	45	41	1	A
14	Sharpening a pencil	0	4	41	55	C
15	Taking the cap off a bottle	1	21	76	1	B
16	Spreading butter on a slice of bread	1	27	72	0	A
17	Fastening a snap (jacket, bag)	3	12	84	1	B
18	Buttoning up trousers	4	29	65	1	B
19	Opening mail	0	6	84	0	B
20	Pulling up the zipper of trousers	2	16	80	1	A
21	Squeezing tooth-paste on a toothbrush	0	8	92	0	A
22	Unwrapping a chocolate bar	1	32	63	4	A
23	Washing one’s hands	0	5	95	0	A

Items ranked from 1 to 23: from more difficult to less difficult. *NA* not applicable. Level A: the item is breakable into unimanual sequences; level B: the item requires stabilization with the affected limb; level C: the item requires fine bimanual dexterity. Due to truncation the percentage values do not add up to 100 % for all items

### Perceived ability to perform daily hand activities and associated factors

Table 3 presents a summary of data regarding perceived ability to perform daily hand activities and the potentially associated factors (i.e., pain and spasticity in the more affected upper extremity; grip strength, active touch and dexterity in the more affected hand; perceived participation and life satisfaction).

**Table 3:** Summary of the measurements (*n* = 75)

Daily hand activities (ABILHAND logits), mean (SD)	2.0 (SD 1.7)
Upper extremity pain (present), n (%)	32 (43)
Spasticity (present), n (%)^a^	23 (31)
Grip strength (newton), mean (SD)^b^	198 (110)
Active touch (0 to 6 points), mean (SD)/median (min-max)^c^	3.8 (2.2)/5 (0 to 6)
Dexterity (0 to 12 points), mean (SD)/median (min-max)^d^	5.4 (3.3)/5 (0 to 12)
Participation (0 to 100 %), mean (SD)/median (min-max)^e^	69 (19)/67 (12 to 100)
Life satisfaction (dissatisfied), n (%)^f^	35 (47)

Data obtained by: ^a^the Modified Ashworth Scale; ^b^the Grippit dynamometer; ^c^the Shape/Texture Identification Test; ^d^the modified Sollerman Hand Function Test; ^e^the Stroke Impact Scale domain Participation; and ^f^the Life Satisfaction Questionnaire (life as a whole)

#### Correlation analyses

Table 4 shows the bivariate correlations among the potentially associated factors treated as continuous or ordinal variables. The correlations were generally low (rho < 0.5) except for the correlation (rho = 0.68) between dexterity and active touch (somatosensation).

**Table 4:** Bivariate correlations (rho) between ordinal and continuous factors (*n* = 75)

	Age	Grip strength	Active touch	Dexterity
Grip strength	−0.21^a^			
Active touch	−0.03	0.24^a^		
Dexterity	−0.19	0.46^b^	0.68^b^	
Participation	−0.18	0.24^a^	0.21	0.21

^a^Correlation is significant at the 0.05 level

^b^Correlation is significant at the 0.01 level

#### Univariate regression analysis

Table 5 presents the univariate associations between perceived ability to perform daily hand activity (logits) and potentially associated factors obtained from the univariate linear regression models. The factor that showed the strongest association with perceived ability to perform daily hand activities was dexterity (*R* ^2^ = 0.39, *p* < 0.001). A one unit increase in dexterity corresponded to 0.32 increased logits of the ABILHAND score (i.e., a β-coefficient of 0.32). The associations between age, social situation, vocational situation, spasticity, grip strength, active touch, perceived participation and life satisfaction, respectively, and perceived ability to perform daily hand activities fulfilled the criteria for being included in the multivariate analyses (i.e., *p*-values ≤ 0.20).

**Table 5:** Results from the univariate linear regression models (*n* = 75)

Determinants	*R* ^2^	*p*-value	β-value (95 % CI)
Age (per year increase)	0.04	0.11	−0.04 (−0.09 to 0.01)
Gender (female *vs* male [ref])	0.00	0.60^#^	−0.23 (−1.10 to 0.63)
Social situation (living together *vs* living alone [ref])	0.04	0.07	0.78 (−0.10 to 1.62)
Vocational situation (working *vs* not working [ref])	0.04	0.11	0.82 (−0.19 to 1.82)
Affected hand (dominant *vs* non-dominant [ref])	0.01	0.50^#^	0.26 (−1.04 to 0.51)
Pain (present *vs* not present [ref])	0.01	0.51^#^	−0.26 (−1.04 to 0.52)
Spasticity (present *vs* not present [ref])^a^	0.08	0.01	−1.04 (−1.84 to −0.23)
Grip strength (newton, per 10 units increase)^b^	0.17	<0.001	0.06 (0.03 to 0.09)
Active touch (per unit increase)^c^	0.23	<0.001	0.37 (0.21 to 0.52)
Dexterity (per unit increase)^d^	0.39	<0.001	0.32 (0.24 to 0.41)
Participation (per 10 units increase)^e^	0.19	<0.001	0.39 (0.20 to 0.57)
Life satisfaction (satisfied *vs* dissatisfied [ref])^f^	0.09	0.01	0.98 (0.24 to 1.72)

^#^The variable did not fulfil the criteria, *p* ≤ 0.20, for being included in the continued multivariate evaluations; ref: reference group (indicate the category to which the other category is compared). Data obtained by ^a^the Modified Ashworth Scale; ^b^the Grippit dynamometer; ^c^the Shape/Texture Identification Test; ^d^the modified Sollerman Hand Function Test; ^e^the Stroke Impact Scale domain Participation; and ^f^the Life Satisfaction Questionnaire (life as a whole)

#### Multivariate regression analyses

Table 6 presents the three factors that were associated with the perceived ability to perform daily hand activity (logits) in the final multivariate linear regression model: dexterity (*p* < 0.001), perceived participation (*p* = 0.002) and grip strength (*p* = 0.180). The explained variance was 39 % for dexterity, which increased to 48 % when perceived participation was added to the model. Grip strength only increased the explained variance with 1 %, but was added due to the generous inclusion criterion (*p* ≤ 0.20). The β-coefficient for dexterity changed from 0.32 in the univariate model to 0.26 in the multivariate model and the corresponding change for perceived participation (per 10 units increase) was 0.39 to 0.26.

**Table 6:** The final multivariate linear regression model (*n* = 75)

Factors	β-value (95 % CI)	*p*-value	Explained variance^d^ (%)
Dexterity (per unit increase)^a^	0.26 (0.16 to 0.35)	<0.001	
Participation (per 10 units increase)^b^	0.26 (0.10 to 0.42)	0.002	
Grip strength (newton, per 10 units increase)^c^	0.02 (−0.01 to 0.05)	0.180	
Dexterity			39
Dexterity + participation			48
Dexterity + participation + grip strength			49

Data obtained by: ^a^the modified Sollerman Hand Function Test; ^b^the Stroke Impact Scale domain Participation; and ^c^the Grippit dynamometer. ^d^Explained variances after successive addition of determinants

## Discussion

The main findings of this study were that bimanual tasks requiring a high level of fine bimanual dexterity were perceived most difficult to perform. Dexterity was the factor that had the strongest association with perceived ability to perform daily hand activities and explained together with perceived participation 48 % of the variance in the final multivariate model.

### Description of perceived ability to perform daily hand activities

The ratings of the items in the ABILHAND revealed that eight of the 23 items were perceived difficult or impossible to perform. All of those items were bimanual tasks classified as requiring a high level of fine manual dexterity (level C) [4] except for the item ‘*buttoning up a shirt*’. Many of the participants expressed that they could manage the buttons on the chest, but the ones on the sleeves were difficult or impossible to perform with the more affected hand. Thus, even if this item is classified as breakable into unimanual sequences (level A) this suggests that it actually requires fine manual dexterity of the more affected hand (level C). Moreover, the item ‘*peeling potatoes*’ is classified as a task requiring fine bimanual dexterity (level C) [4] but was considered as easy for a majority of the participants in our study. Peeling potatoes in Sweden is normally done with a special potato-peeling tool instead of a knife that is suggested in the original version of the questionnaire [4], and this could possibly explain why this item was rated as easy. The other items that were rated as easy were primarily breakable into unimanual sequences (level A) or only required stabilization with the affected upper extremity (level B). As ability to perform daily hand activities is an important goal in the rehabilitation after stroke [5] the ratings of the items in the ABILHAND could be helpful for the patients in their goal setting. Moreover, the different levels (A, B and C) may be useful for the clinicians in the analysis of daily hand activities regarding the bimanual involvement and dexterity demands. The underlying sensorimotor functions required for the tasks can then be specifically practiced in order to achieve the patient’s goals.

### Perceived ability to perform daily hand activities and associated factors

In the present study, the association between perceived ability to perform daily hand activities and several factors were analysed. All factors, except for upper extremity pain, gender and affected hand, were included in the multivariate analyses. Dexterity of the more affected hand was strongest associated with perceived ability to perform daily hand activities. Manual dexterity includes the ability to execute coordinated hand and finger movements when grasping, manipulating and releasing objects. Reduced dexterity can result in impaired grip formation and independent finger movements, and in reduced timing and force regulation of the hand [38, 39]. As manual dexterity is important for upper extremity functioning it should be thoroughly assessed and intensively practiced during stroke rehabilitation to enhance the use of the hands in daily activities.

In the final multivariate model, perceived participation was included as the second strongest factor and added another 9 % of the explained variance. This underscores the importance of considering a person’s engagement in meaningful life situations in stroke rehabilitation. Traditionally, most of the rehabilitation is carried out in the early phase after stroke. However, it has been shown in a recent large European multicenter study that the arm function and activity performance for persons with stroke deteriorate over time [40]. This indicates that longer follow-up periods and interventions in the later phases after stroke are needed, with emphasis on regaining and finding new ways to participate in meaningful activities in order to maintain the ability to perform daily hand activities over time.

Furthermore, in our final multivariate model grip strength was included as the third factor, but added only 1 % to the explained variance. In the study by Harris and Eng [14] both arm and grip strength were associated with the ability to perform daily hand activities. Arm strength was the strongest contributor in their final multivariate model and together with spasticity and grip strength they explained 93 % of the variance in daily hand activities. In the present study, we used grip strength as a proxy for muscle strength of the entire upper extremity, as grip strength has been shown to be highly correlated to shoulder and elbow muscle strength after stroke [27]. Dexterity was not included in the study by Harris and Eng as they included persons with more severe impairments in the upper extremity after stroke. Thus, our results are difficult to fully compare with that study. Altogether though, the findings indicate that manual dexterity is more important for daily hand activities in persons with milder impairments, whereas strength is more important for persons with severe impairments.

Despite that we included several potentially associated factors in our multivariate analyses, dexterity and perceived participation were the only significant contributors in the final model and together they explained 48 % of the variance of perceived ability to perform daily hand activities. Even though this is a high degree of association, it still suggests that other factors may be important to consider in the rehabilitation of persons with mild to moderate impairments of upper extremity after stroke and that daily hand activities need to be measured and trained *per se*.

Somatosensation (i.e., active touch) was the factor that showed the second highest correlation with perceived ability to perform daily hand activities. However, active touch was not included in the final multivariate model due to the high correlation with dexterity. This suggests that to be able to perform manual dexterity tasks somatosensation is an important underlying factor. Somatosensation, measured as passive touch, has in a previous study [41] been found to correlate significantly with dexterity in persons with mild impairments in the upper extremity after stroke. Future studies should therefore investigate how different modalities of somatosensation are associated with gross and fine manual dexterity and the influence on the performance of daily hand activities after stroke.

### Strengths and limitations

A strength of the present study was that the participants with mild to moderate upper extremity impairments after stroke were recruited from an unselected population living both in urban and non-urban areas in the southern part of Sweden. The participants were all in a stable phase post-stroke, care was taken to standardize the test situation and one examiner performed all assessments. Stroke specific outcome measures were used to assess functioning and disabilities of the upper extremity, valid and reliable for persons with stroke. Moreover, the multivariate model included several factors in order to evaluate their association with perceived ability to use the upper extremities in daily activities after stroke.

The sample size in the present study was based on a reasonable number of persons judged to be sufficiently large to evaluate the associations of interest. However, for weak associations a larger study sample is required. Only persons with mild to moderate impairments of upper extremity were included in the present study, and those with major cognitive impairments or difficulties to communicate were excluded. More men volunteered to participate and the results can therefore not be generalized to the entire stroke population. Furthermore, subgroup analyses to evaluate differences regarding gender, dominant or non-dominant affected hand and time since stroke would have been interesting, but were not conducted as we judged the sample size being too small. Moreover, it cannot be excluded that other factors may be of importance for the ability to perform daily hand activities after stroke, for example vision, cognitive functions, fatigue, self-efficacy, aids, family and health care support. These variables were not included in the present study as too many variables may cause fatigue in the assessments of persons with stroke.

As this was a cross-sectional study we cannot state that the causality directly results from the factors included in the regression models. Prospective studies are therefore needed in order to evaluate how several potentially associated factors influence the perceived ability to perform daily hand activities in persons with mild to moderate impairments of upper extremity after stroke over time.

## Conclusion

This cross-sectional study showed that the daily hand activities that were perceived difficult or impossible to perform were tasks requiring a high level of fine bimanual dexterity. Dexterity was the strongest contributor to perceived ability to perform daily hand activities and together with perceived participation explained 48 % of the variance in the final multivariate model. This suggests that dexterity and participation are particularly important to consider in the rehabilitation of upper extremity after stroke. The explained variance implies that other factors may also be of importance to improve the ability to use the hands in daily life after stroke.

## Abbreviations

CI: Confidence interval
LiSat-11: Life Satisfaction Questionnaire
MAS: Modified Ashworth Scale (MAS)
mSHFT: modified Sollerman Hand Function Test
SD: Standard deviation
SIS: Stroke Impact Scale
STI-test: Shape/Texture Identification test
